# Leveraging LLM to identify missed information in patient-physician communication: improving healthcare service quality

**DOI:** 10.3389/fmed.2025.1631565

**Published:** 2025-08-01

**Authors:** Xingyou Zhou, Eldan Cohen, Xingzuo Zhou, Enid Montague

**Affiliations:** ^1^Department of Mechanical & Industrial Engineering, University of Toronto, Toronto, ON, Canada; ^2^Institute for Global Health, University College London, London, United Kingdom

**Keywords:** large language model (LLM), automation, patient satisfaction, patient safety, missed information

## Abstract

**Background and objective:**

Electronic medical records (EMRs) have significantly changed the dynamics of physician-patient interactions, leading to a shift in communication patterns. Although various studies have developed guidelines for these new dynamics, different EMRs result in different modes of interaction, which can contribute to missed information during clinical encounters. Therefore, this study aims to develop a method that can automate the identification process of missed information to increase patient safety and satisfaction.

**Methods:**

A total of 98 transcripts of clinical consultations from two primary care clinics in the United States were used for identifying missed information and patient unsatisfactory factors. We first examine those factors through ordinal logistic regression. Then we leveraged large language model (Phi-3.5) to develop the automation model for identifying missed information of physicians.

**Results:**

We show that showing care and empathy to patients ( β =1.283, OR = 3.609 [95% CI: 1.836, 7.091], p <0.001) and explaining things clearly to patients ( β =1.620, OR = 5.051 [95% CI: 2.138, 11.938], p <0.001) can significantly increase the level of patient satisfaction. And our model has an average accuracy of 90.09% with F1-score of 93.75% on identifying missed information during clinical practices in primary care.

**Conclusion:**

This study demonstrates the potential of automated analysis using Phi-3.5 to evaluate the identification of communication gaps in physician-patient interactions, ultimately enhancing patient safety and satisfaction. Further research is needed to refine this approach and explore its application across diverse healthcare settings.

## 1 Introduction

New technologies in healthcare are designed to assist physicians to provide healthcare services with higher efficiency which have changed the visiting patterns permanently for both physicians and patients (1, 2). For example, the use of Electronic Medical Records (EMR) saves time for physicians spending time on communication with patients so that they can take care of more patients (3, 4). However, the increasing use of EMRs and other computer-based systems in primary care has added complexity to physician-patient interactions. While these technologies have numerous benefits, including improved record-keeping and data accessibility, they can also be a source of distraction for physicians. The workload of the physicians has also increased due to the increasing number of visits (5). Studies have shown that physicians spend a significant portion of their time interacting with computers during patient visits, which can distract from communication with the patient (6, 7).

Diagnostic errors in primary care are a significant concern, with studies indicating that they occur in approximately 5 to 15% of encounters, depending on the conditions examined and the study parameters (8, 9). Essential information during the clinical practices can be missed by communication breakdowns between physicians and patients which leads to the result of incorrect diagnosis (10). Physicians may miss information from patients, including verifying personal information in health records with them and gathering details of their medical history, medication use, and findings from physical and medical examinations (11, 12). Increased computer usage has raises concerns about its impact on the quality of care and the potential for missing crucial patient information (13).

As the new technology of Artificial Intelligence (AI) is deploying in the healthcare system, the physicians are able to manage with much more information as before and take care of more patients (14–16). Since physicians have less communications with patients, the new type of communication pattern is emphasized since the period of using of EMR that the patients are encouraged to join the diagnosis process during the visiting with more active interactions with physicians instead of being passive (17–19). The quality of the communication between the physicians and patients is extremely important during this process, as any pieces of missed information will lead to the incorrect diagnosis and decision-makings of physicians.

Patient satisfaction and trust are also significant factors for studying communication patterns in healthcare. Although new healthcare technologies have increased efficiency of physicians. Reduced direct communication with patients often leads to perceptions of decreased attentiveness and care, resulting in lower patient satisfaction and weakened trust in the healthcare experience. Previous studies shows that the lack of interactions between physicians and patients will lead to the feeling of insufficient care for patients (19, 20). In particular, it is essential to balance the use of new technology with strategies to maintain patient-centered communication to ensure the quality of healthcare service provided and preserve the trust in the physician-patient relationship.

Therefore, it is necessary to study communication patterns and effectiveness to uncover factors that contribute to suboptimal healthcare services and instances of incorrect diagnosis (21). To identify the causes of incorrect diagnoses, previous studies have used an event-based reporting system to identify the information physicians may have missed from patients. This system is structured to monitor specific events that could potentially lead to the incidents (22, 23). However, the unique nature of primary care presents challenges for this system. In primary care, the scope of interaction is much more complex and broader than in specialized departments, such as emergency department, where primary care emphasizes a long-term, continuous relationship between physicians and patients, with comprehensive, ongoing conversations (10, 24). Hence, instead of focusing solely on critical events in emergency or surgical settings, routine patient–physician interactions are recognized as key factors that physicians need to prioritize in primary care (25, 26). And physicians in primary care are tasked to understand and address the concerns and needs from patients (27, 28). That is, patient-centered care is valued more by patients in primary care than in emergency department settings, identifying key drivers that influence the quality of interactions during clinical practice is significant (29).

Traditional methods such as manual coding are widely used to study behavior and communication patterns during clinical encounters (26, 30, 31). However, these methods present significant challenges in terms of cost and efficiency, making it difficult to analyze large datasets. As the advancements in artificial intelligence, machine learning and natural language processing approaches are increasingly being used for automating event analysis (32, 33). With the development of deep learning techniques, particularly through transformer-based Large Language Models (LLMs), conversation dialogs can be effectively modeled and understood with the technique of attention mechanism in transformers (34–36). Hence, we are able to analyze complex communication patterns and capture events from a conversation by considering full context of the text, including the sequence of messages and underlying meanings across the turnovers (37–40). This capability presents an opportunity to analyze information may missed during conversations in primary care while interactions and turnovers during primary care clinical practices are centralized with immediate solutions and reactions from physicians to patients (41, 42).

Therefore, this study first aims to identify the missed information from physicians in primary care and reveal factors that affect the quality of patient-centered care during clinical encounters, with a focus on their impact on patient satisfaction. Next, we develop a framework with large language model (LLM) to automate the identification of missed information from physicians, with a goal of increase patient safety in primary care clinics. Lastly, we discussed the unique nature of the missed associated with incorrect diagnosis and the needs of patients in order to provide a solution for increasing their satisfaction with physicians.

## 2 Materials and methods

### 2.1 Data collection and processing

The data used for this study was collected from previous studies which included 110 medical encounters recordings with high enough quality and the survey (31). The protocols of this study and previous studies were received and approved by Research Ethics Boards (Protocol #: 00045360) and clinicians consented to participation. Two clinical centers in the US participated in this study, eligible patients must have infected with common cold. There are 110 patients, and 5 physicians participated in this study, including 41 males and 69 females. And the mean age of patients was 34.2 (min 12.2, max 71.8). Eligible patients who agreed or authorized by guardian to participate were taken to a private consultation room with video cameras and a survey was filled out by each patient after the consultation. In the survey questionnaire, each patient was asked to rate their satisfaction with their physicians, and the level of showing care (i.e., needs, compassion) and explaining things clearly to patients. There are 5 levels of the rating, from 1 to 5 for each category.

**Figure 1:**
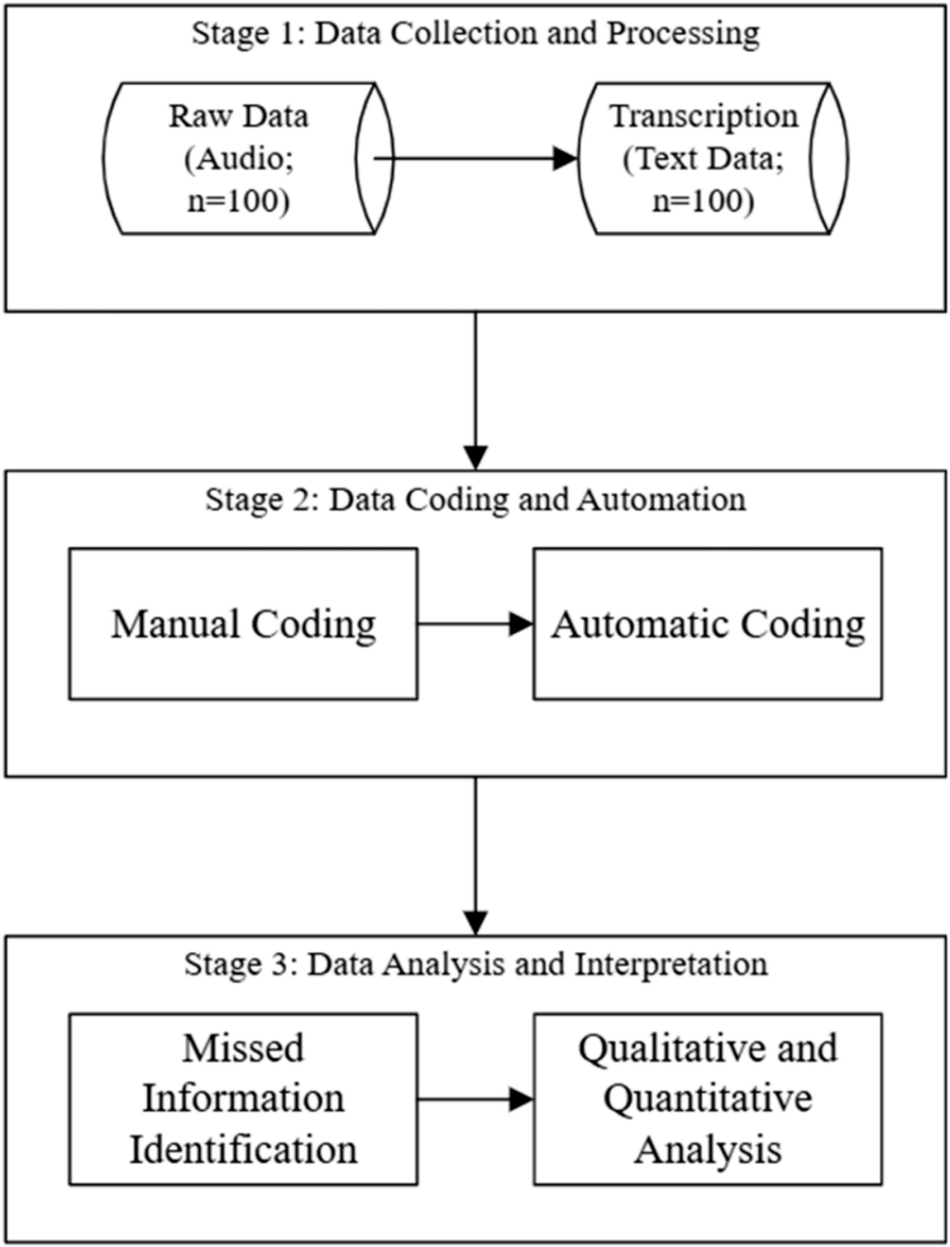
Flowchart for this study.

The audio data were extracted from each encounter. Following Research Ethics Board (REB) guidelines, we used offline versions of the python programs. Transcriptions were obtained using whisper (43), and speakers were identified using pyannote.audio (44). Each transcription of an encounter was saved into a CSV file for analysis. A flowchart of the data processing process is shown in Figure 1.

### 2.2 Clinical encounters and patient satisfaction

**Figure 2:**
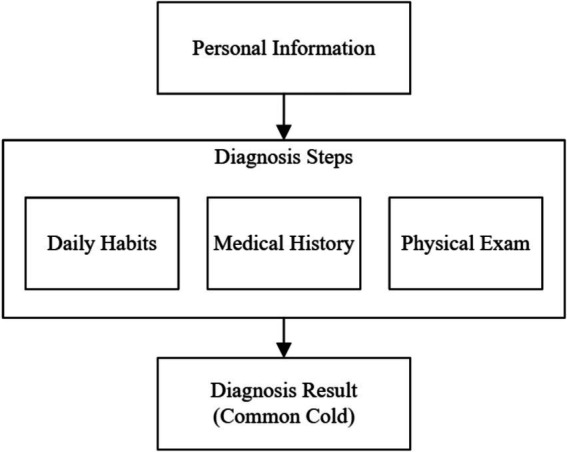
Framework of identifying missed information and diagnosis procedures.

**Figure 3:**
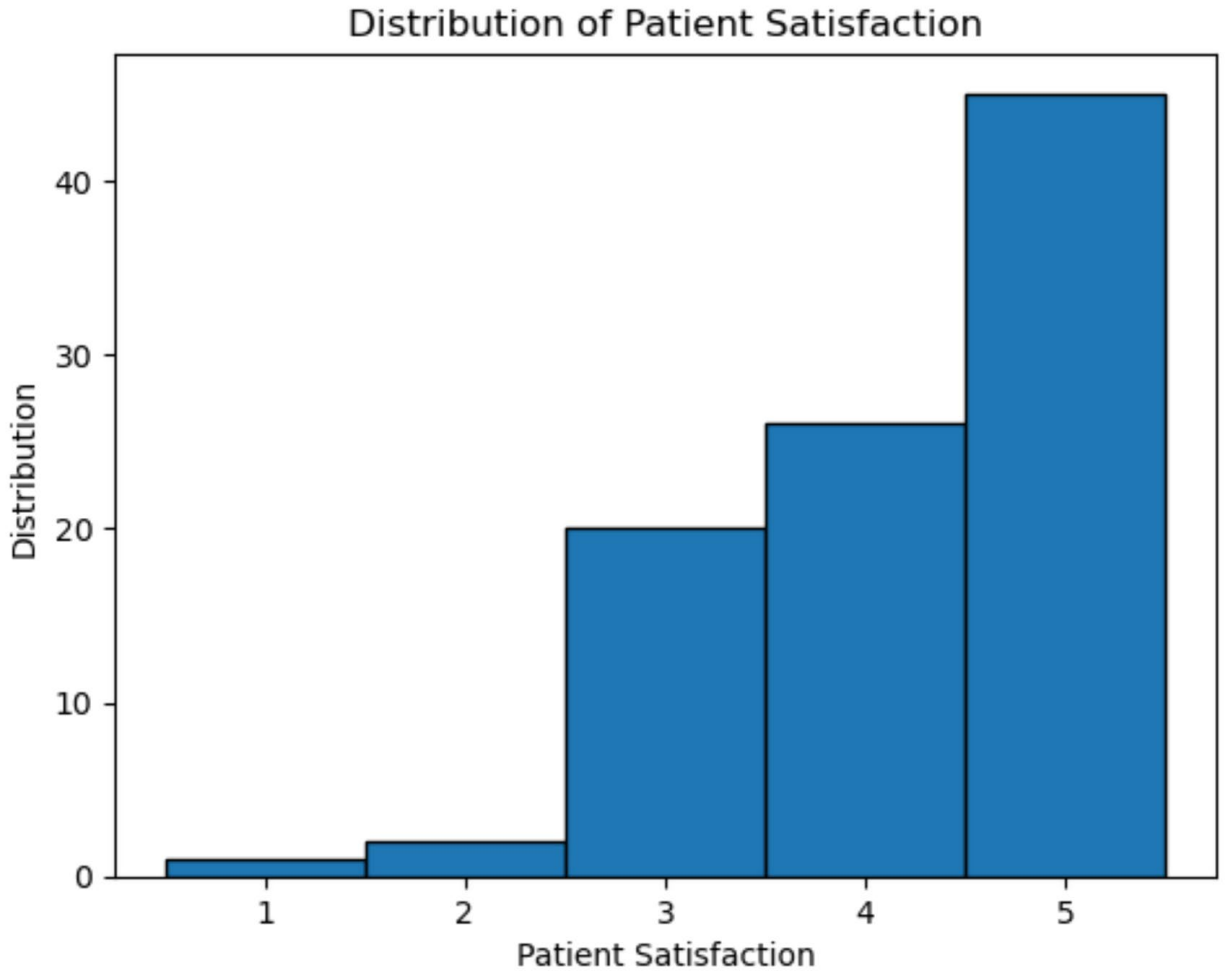
Distribution of patient satisfaction.

In the diagnostic steps for the common cold in primary care, physicians often perform physical examinations to identify symptoms and make a diagnosis (45). Studies have shown that most cases of the common cold are caused by rhinoviruses, and symptoms such as rhinorrhea, nasal congestion, and sore throat are typically identifiable through a physician’s examination, around 20% of cold cases are caused by unknown viruses (46). These cases may require laboratory tests to guide treatment effectively. However, physicians often avoid lab testing during initial visits due to the defensive healthcare practices, where simpler diagnoses are prioritized to reduce patient expenses and avoid unnecessary interventions (47, 48). In primary care, medical examinations are not required for the common cold unless symptoms have lasted more than typical duration or atypical symptoms are present (45). Therefore, based on guidance from previous studies, we developed a framework designed to identify missed information of diagnostic process toward patients performed by physicians, with the aim of increasing patient safety and satisfaction (45, 46, 49). A framework of our model on identifying missed information and procedures in clinical encounters is shown in Figure 2, with the explanation of each stage provided in Table 1. In addition, all participating physicians in this study made accurate diagnoses, and the annotation of missed information in each diagnostic step was performed by a single annotator (XZ) through a manual review of the dialog text of the encounters. And we selected showing care, explaining things clearly to patients and their satisfaction levels in the survey data (each factor is rated by 1 to 5) collected by Osan and Montague as factors in examine patient centered care to patient satisfaction (26, 50, 51). The distribution of the diagnostic process and patient centered care factors is provided in Table 2. Figure 3 is the distribution of patient satisfaction levels.

**Table 1:** Explanation of framework.

Personal information	Physicians check in patients for their appointments and verify their personal information, which is verified before the experiment by nurse.
Diagnosis Steps: Daily Habits	One of the diagnostic steps; Physicians check daily habits of patients may cause symptom of common cold (i.e., alcohol, tobacco use; 1 = Yes, 0 = No)
Diagnosis Steps: Medical History	One of the diagnostic steps; Physicians check medical histories of patients (i.e., symptoms, allergies, genetic diseases; 1 = Yes, 0 = No)
Diagnosis Steps: Physical Exam	One of the diagnostic steps; Physicians perform physical exams for patients (i.e., Temperature, Oropharyns, Nose, Chest, Neck stiffness; 1 = Yes, 0 = No)
Diagnosis Result (Common Cold)	The diagnosis results of our data are common cold, which is selected (controlled) during the data collection.

**Table 2:** Distribution of diagnostic process and patient centered care factors.

Diagnostic process (completion rate)		Patient centered care			
Daily habits	81%	ShowingCare(1)	4%	Explaining(1)	1%
Medical history	97%	ShowingCare(2)	8%	Explaining(2)	5%
Physical exam	99%	ShowingCare(3)	13%	Explaining(3)	15%
Overall	81%	ShowingCare(4)	18%	Explaining(4)	21%
		ShowingCare(5)	57%	Explaining(5)	58%

### 2.3 Model specification

In this study, we first implemented an ordinal logistic model to analyze patient satisfaction. The ordinal logistic model accounts for the ordered nature of outcome without assuming equal intervals between different outcome levels (52). That is, the ordinal logistic model allows the analysis of patient satisfaction levels to inherent the order of responses without treating difference between adjacent satisfaction levels as equivalent. Given a response variable $Y_{i}$ with 5 satisfaction levels, the probability of a patient satisfaction for a clinical encounter can be defined as:


$$P(Y_{i}>j)=\frac{\exp(\sum_{k=1}^{K}\beta_{k}X_{\mathrm{ik}}-\alpha_{j})}{1+\exp(\sum_{k=1}^{K}\beta_{k}X_{\mathrm{ik}}-\alpha_{j})},j=1,\ldots ,4$$

where j represents the level of patient satisfaction, $\alpha_{j}$ is the cut-off point (threshold) for the $j^{\mathrm{th}}$ satisfaction level, $\beta_{k}$ is a vector of model parameters, and $X_{\mathrm{ik}}$ is a vector of all independent variables. In particular, the details of all independent variables are provided in Table 3. DurationDays, Age and Education are controlled.

**Table 3:** Explanation of independent variables.

Showing care	Survey to patient; rating of showing care (i.e., needs, compassion) to patients (1–5)
Explaining	Survey to patient; rating of explaining things clearly to patients (1–5)
Steps	Number of diagnostic steps has performed by physicians (0–3).
Duration days	Number of days of the symptoms of common cold of patients has been lasted (in days)
Age	The age of patients (in years)
Education	Education level of patients (0 = does not have college/university diploma, 1 = have college/university diploma)

Then we used LLM to automize the manual qualitative coding of missed information during the clinical encounters. The Large Language Model (LLM) is a type of deep learning model designed to understand, generate, and process human language. It is built on transformer architectures, which allows the model to capture complex patterns in text through self-attention mechanisms (34, 53). It can do several tasks effectively, such as text generation, summarization and natural language understanding (37, 38).

In this study, we aim to identify the missed information from the interactions between physicians and patients. The LLM we used for this study is Phi-3.5-mini-instruct (Phi3.5) (54), a relatively small language model designed for faster inference, lower computational resource requirements, and reduced energy consumption. While many large language models, such as Llama3-8B come in different sizes (55), Phi-3.5-mini-instruct is specifically optimized its efficiency, which is more suitable for deployment in local environments.

To improve the performance of the model and increase its understanding of conversations of primary care encounters, we implemented zero-shot in-context learning on the model with the guideline of BMJ Best Practice and a dictionary of epidemiology (45, 56, 57). This allows the model to better recognize and interpret medical terms, improving its accuracy in understanding health-related texts. We first randomly selected approximately 30% of the data as a held-out validation dataset to tune the prompts for the Phi-3.5 model. The rest of data were then used for testing set to evaluate the model’s performance, including accuracy, precision, recall, and F1 score (58). Since our model identifies missed events based on a framework based on a “checklist,” we evaluate its performance by comparing the accuracy between predicted label and actual label on information collected by physicians, and compute precision, recall, F1-score are computed for the clinical encounters in testing dataset.

## 3 Results

### 3.1 Patient centered care

To examine the potential factors influencing patient satisfaction, an ordinal logistic model was fitted to our data. The estimates of the fitted model are shown in Table 4. According to previous studies (51, 59), we selected showing care, explaining things clearly, and the number of diagnostic steps performed by physicians as explanatory variables, and included duration of common cold (in days), age, and education level of patients as control variables. The result shows that showing care to patients ( β =1.283, OR = 3.609 [95% CI: 1.836, 7.091], p <0.001) and explaining things clearly to patients ( β =1.620, OR = 5.051 [95% CI: 2.138, 11.938], p <0.001) can significantly increase the level of patient satisfaction. And following proper diagnostic steps does not have impact toward patient satisfaction. However, this may be attributed to the distribution of our data (Table 2), as most of the information missed during the clinical encounter was checking patients’ daily habits, whereas physicians performed well in other diagnostic steps. That is, we can only show that missed information in daily habits does not affect patient satisfaction.

**Table 4:** Ordinal logistics model regression results.

Variables	Estimates (Model 1)	Odds ratio (95% CI)	Estimates (Model 2)	Odds ratio (95% CI)
Showing care	1.361*** (0.340)	3.900 (2.001, 7.598)	1.283*** (0.345)	3.609 (1.836, 7.091)
Explaining	1.553*** (0.427)	4.723 (2.045, 10.910)	1.620*** (0.439)	5.051 (2.138, 11.938)
Steps	0.533 (0.633)	1.704 (0.493, 5.892)	0.382 (0.661)	1.465 (0.401, 5.351)
Duration days	-	-	−0.033 (0.071)	0.968 (0.843, 1.111)
Age	-	-	0.023 (0.019)	1.023 (0.986, 1.062)
Education	-	-	−0.226 (0.512)	0.798 (0.292, 2.179)

*p < 0.1, **p < 0.05, and ***p < 0.01; robust standard errors are presented in parentheses.

### 3.2 Automation of identifying missed information during diagnostic process

We then automated the identification of missing information in clinical encounters by utilizing the LLM (Phi3.5) for improving patient safety during the diagnostic process. Rather than monitoring for missed events, we counted the events that occurred (22, 60). In this context, a positive label indicates that the information was not missing. Additionally, a total of 98 recordings were used for this study, 12 recording were excluded due to low audio quality.

Since the data set was positively skewed (i.e., missed information was rare in clinical encounters) we first generated 50 synthetic clinical encounters. In particular, interactions within each synthetic encounter were randomly selected from original data and combined to form a coherent and realistic conversation with missed information during random diagnostic process. We then applied zero-shot learning using Phi-3.5 (57), with 40 encounters (25 actual and 15 synthetic) held out as validation set for prompt tuning on Phi-3.5, and 108 encounters (73 actual and 35 synthetic) as the test set. The prompt for zero-shot learning is provided in Appendix. The automated identification model performance is presented in Table 5. Although our model has lower accuracy (0.77), recall (0.78) and F1-score (0.85) in DailyHabits than other categories (>0.95). We can identify missed events very well since low recall in DailyHabits means that there are many times the doctor actually did collect information about daily habits, but the model failed to detect it. In overall, our model shows strong performance in capturing the events occurs (58), which means that our model can identify missed information during clinical encounters with a high performance.

**Table 5:** Performance table of automated identification model.

Variables	Accuracy	Precision	Recall	F1-score
Daily habits	0.7703	0.9216	0.7833	0.8468
Medical history	0.9595	0.9595	1	0.9793
Physical exam	0.973	0.9863	0.9863	0.9863
Average	0.9009	0.9558	0.9232	0.9375

## 4 Discussion

This study explored the impact of missed information during clinical encounters on patient safety and satisfaction in primary care using samples from university primary care clinics in the US. To identify missed information in clinical encounters, we categorized the essential diagnostic information for assessing the common cold during primary care encounters into three domains: daily habits, medical history, and physical examination. Our analysis of missed information reveals that physicians failed to collect information related to daily habits of patients in 19% of the clinical encounters. Although missed information from physicians does not show a significant effect on diagnostic outcomes in this study, this may be attributed to the imbalanced distribution on missed information, previous studies have indicated that missed information is one of the significant factors contributes to errors in primary care settings, which can lead to incorrect diagnoses (8, 9). For instance, missed information in patients’ daily habits can have various negative consequences that missing smoking habits of patients with respiratory symptoms may result in poorer disease control, increased healthcare utilization, and higher healthcare costs (61), and missing information of patients’ alcohol consumption habits may result in adverse health outcomes, particularly when medications that interact negatively with alcohol are prescribed (62).

Notably, although this study did not directly examine the impact of EMR use on missed information, previous research has shown that physicians who are proficient with electronic medical records are less likely to miss critical information during clinical encounters, as they need to input and retrieve data through the EMR system (3, 26). Furthermore, the increasing use of EMRs and emerging AI tools in clinical practice can help reduce likelihood of missed information by providing more intuitive and supportive interfaces for physicians through EMR system (4, 18). These technologies can also save time, particularly under conditions of high workload, by minimizing the need to repeatedly collect the same patient information across clinical visits (45, 63). However, workload of physicians may paradoxically increase with the use of more efficient EMR systems, particularly in the context of physician shortage (3, 64). One consequence of increased workload is burnout of physicians, which has been associated with a higher likelihood of missed information where essential information may be ignored during clinical decision-making and diagnosis (7).

With respect to patient satisfaction, our data show a high proportion of responses indicates high satisfaction levels. One significant factor contributes to these ratings appears to be the effective documentations by physicians (26, 30, 31). The ability of physicians to provide attentive care while efficiently doing documentation may enhance patients’ trust and overall satisfaction. The results of the ordinal logistic regression analysis on attentive care reveal similar findings that when physicians demonstrate compassionate care and provide clear explanations to patients, it significantly increases patient satisfaction. Our ordinal logistic regression analysis does not show a statistically significant correlation with missed information and patient satisfaction. One of the reasons is that physicians are the primary source of information for patients (19). That is, patients are likely to trust the diagnostic process even in cases where errors occur or information is missed and satisfy with physicians when they show compassionate and effective communication skills (19–21). However, this may vary across different clinical settings, particularly the encounters with longer waiting times. Waiting time has been identified as a significant factor contributing to lower patient satisfaction (64, 65). Key determinants of patient satisfaction include the amount of time spent with physicians (65) and patients’ perceptions of being treated attentively and respectfully during clinical visits (64). In particular, when patients experiencing extended waiting times, they expect more comprehensive diagnostic procedures than their previous visits.

As our goal is to examine the impact of missed information during clinical encounters on patient safety and satisfaction using a larger sample in future studies, analyzing the interactions between physicians and patients through manual annotation presents substantial challenges. To address this challenge, we developed a method for automatically identifying missed information using a large language model (LLM), specifically Phi-3.5. Our model demonstrated strong performance in identifying missed information across all diagnostic steps (F1-score > 0.8) by using the designed prompt to LLM (57, 58). In addition, we found that identifying missed information in daily habit (F1-score = 0.8468) has slightly lower F1-score than other categories (F1-score > 0.97), the challenge may arise by the difficulty of collecting all relevant habits that are involved. For instance, we have only included most common habits related to the diagnosis of the common cold in this study (45). However, some physicians are interested with specific habits, such as coffee consumption, which were not considered in our model. This discrepancy can lead to incorrect coding during the identification of missed information, where physicians may have documented that certain habits were assessed, but our model lacks the context to recognize the relevance of these specific habits.

This study has several limitations that should be considered. Although previous research has demonstrated that missed information have negative impact on patient safety and satisfaction, our study did not replicate these findings, primarily due to a limited sample size. In particular, the dataset lacked sufficient cases involving incorrect diagnoses, which restricted our ability to examine the specific effects of missed information on diagnostic accuracy and patient satisfaction. However, this study is still valuable for future works. Our Phi3.5 model can classify the information during clinical practice with high performance. That is, we provided an effective method to analyze with larger sample sizes on missed information.

Furthermore, future studies should consider incorporating additional data sources, such as using EMR usage logs during clinical practices to identify the information retrieved from EMR system by physicians when patients have previously visited (4, 26). Video recordings of clinical encounters could provide valuable insights into physician behavior and cognitive load, particularly in their burnout. Physicians experiencing burnout during work may fail to properly process or apply the information available to them, even when it is accessible (7). Also, body language can be analyzed through video data, as nonverbal communication has been shown to significantly influence patient satisfaction (26, 31). In addition, a standardized scaling system for assessing ratings of physician empathy and explanatory communication should be implemented. This would enable more consistent evaluation of patient-centered care factors, potentially allowing for sentiment analysis using language models (26, 30, 54).

## 5 Conclusion

Our study provides evidence that improving interactions during clinical practices receive higher satisfaction from patients. The method regarding LLM we provided with, have contribute to the automated analysis on missed information in clinical interactions, ultimately enhancing patient safety by preventing incorrect diagnosis. It demonstrates potential capabilities in information identification through a multimodal approach. Future studies should examine diverse healthcare settings to ensure that the proposed framework can better align with the essential information needs of clinical practice. Additionally, technologies such as EMRs in primary care should be designed to strengthen the connection between physicians and patients, not only by improving information transparency for both sides, but also by better accommodating user preferences.

## Data Availability

The datasets presented in this article are not readily available because original data include recording of clinical practices, which can identify the participants. Requests to access the datasets should be directed to enid.montague@utoronto.ca.

## Appendix

In this study, we applied prompt engineering with Phi3.5, with the following prompts:

Here are examples of diagnostic steps:

1. A history eliciting a constellation of symptoms compatible with the diagnosis
2. Identification of risk factors suggestive of the condition (for example, seasonal occurrence, smoking, exposure to affected individuals)
3. A brief physical examination, including temperature, pulse and blood pressure, and examination of oropharynx, nares, neck, and chest. If the patient is unwell or observations are outside of normal limits, consider other causes or complications such as influenza serious bacterial infections such as pneumonia or meningitis, or sepsis, and tailor physical examination accordingly
4. Excluding alternative diagnoses by screening for distinguishing features of conditions with overlapping symptoms, such as allergic rhinitis.

The following is a conversation between a physician and a patient in a clinical encounter that patient infect with common cold (Speaker 1: Physician, Speaker 0: Patient, Speaker U: Nurse/Staff):

{Conversation dialog}.

Please summarize the following into ‘Yes’ or ‘No’ answers (If patient shares the information before physician asks, it also counts as yes):

1. Did Physician receive information about the daily habits of patients?

- Examples: alcohol, smoke/smoking/tobacco, jogging, etc.

1. Did Physician receive information about the medical history of patients?

- Examples: symptoms (A symptom is any subjective evidence of disease perceived by the patient), allergies, genetic diseases (such as Heart Disease, Asthma or other genetic disease affecting breath) compatible with the diagnosis.

1. Did Physician receive physical exam information from patients?

- Patient provide information about the information or feelings of their temperature, oropharynx, nares, neck, chest
- Physician perform physical exam to patients with instructions such as Now I will check your XXX. (XXX: temperature, oropharynx, nares, neck, chest of patients).
